# Systemic Administration of Orexin a Loaded Liposomes Potentiates Nucleus Accumbens Shell Dopamine Release by Sucrose Feeding

**DOI:** 10.3389/fpsyt.2018.00640

**Published:** 2018-12-03

**Authors:** Francesco Lai, Flavia Cucca, Roberto Frau, Francesco Corrias, Michele Schlich, Pierluigi Caboni, Anna Maria Fadda, Valentina Bassareo

**Affiliations:** ^1^Department of Life and Environmental Sciences, University of Cagliari, Cagliari, Italy; ^2^CNBS, University of Cagliari, Cagliari, Italy; ^3^Department of Biomedical Sciences, University of Cagliari, Monserrato, Italy; ^4^Cagliari Section, National Institute of Neuroscience, Monserrato, Italy

**Keywords:** orexin A, SB 334867, nucleus accumbens, dopamine, shell, sucrose pellets, liposomes

## Abstract

Orexin neurons originate in the lateral and dorsomedial hypothalamus and perifornical area and produce two different neuropeptides: orexin A (OxA) and orexin B (OxB), which activate OxR1 and OxR2 receptors. In the lateral hypothalamus (LH) orexin neurons are involved in behavior motivated by natural rewards such as palatable food (sugar, high-fat food) and it has been demonstrated similarly that the orexin signaling in the ventral tegmental area (VTA) is implicated in the intake of high-fat food. The VTA is an important area involved in reward processing. Given the involvement of nucleus accumbens (NAc) shell dopamine (DA) in motivation for food, we intended to investigate the effect of OxA on the basal and feeding-activated DA transmission in the NAc shell. OxA is a large peptide and does not cross the blood–brain barrier and for this reason was loaded on two kinds of liposomes: anti-transferrin-monoclonal antibodies (OX26-mAb) and lactoferrin-modified stealth liposomes. The effect of IV administration of both OxA liposomes on NAc shell DA was studied by microdialysis in freely moving rats. OxA, administered using both kinds of liposomes, produced a delayed and transitory increase in dialysate DA in the NAc shell, strongly and lastingly potentiated the increase in dialysate DA elicited by sucrose pellet consumption and increased the number of eaten pellets. These effects of OxA on DA transmission and feeding were prevented by the OxR1 antagonist SB 334867. Hence, OxA acting on VTA OxR1 can facilitate sucrose-stimulated NAc shell DA transmission directly by increasing the basal activity of VTA DA neurons that send their projections to the NAc shell.

## Introduction

Many neuronal systems are involved in the regulation of behavioral responses essential for the survival of the individual and of the species. In particular, the dopaminergic mesolimbic system and the orexin system are implicated in several aspects of the rewarding properties of food (1–8).

Orexin neurons are located in the perifornical nucleus, the dorsomedial hypothalamic nucleus, and the dorsal and lateral hypothalamic areas and project widely through the central nervous system (CNS), including the cerebral cortex, the hippocampus, the thalamus, the midbrain, and the spinal cord (9–12).

Orexin neurons produce two peptides from a common pre-proorexin molecule; these peptides are orexin A (OxA) and orexin B (OxB), which are 33 and 28 amino acids in length, respectively. Two distinct G protein-coupled receptors, namely, the orexin-1 receptor (Ox1R) and the orexin-2 receptor (Ox2R), for OxA and OxB have been characterized. OxA shows a 10-fold higher selectivity for Ox1R, and OxB shows equal affinity for both receptors (10, 11). Harris and Aston–Jones (4) highlighted a dichotomy in the functions of orexin neurons. These authors reported that orexin neurons in the lateral hypothalamus are implicated in food responsiveness, while orexin neurons in the perifornical and dorsomedial hypothalamus play an important role in arousal and stress responsiveness.

Several studies have demonstrated the role of the hypothalamic orexin system in the regulation of food intake. Central administration of OxA produces food consumption, preferentially consumption of high-fat food (10, 13–15), which in turn activates dopaminergic neurons in the ventral tegmental area (VTA) and orexin neurons in the lateral hypothalamic area (16). Moreover, OxA is implicated in food-seeking motivated by hunger (17) or by Pavlovian cues (18–20). The OxR1 antagonist, namely, SB 334867, reduces food consumption (21) as well as high fat chocolate and sucrose self-administration (5, 6).

It is well-known that dopamine (DA) plays an important role in the responsiveness to food consumption. Natural rewards, such as food, increase DA release in the nucleus accumbens (NAc) shell (22–25) and this increase undergoes rapid habituation after the first meal of the same food (22, 26–29). The habituation of NAc shell DA response during food consumption is consistent with a facilitatory role of the shell DA in associative learning, when the acquisition of incentive properties by reward predictive stimuli occurs (2, 30–32). We recently reported that the extracellular DA also increases selectively in the NAc shell of rats responding for sucrose pellets by nose poking (33–35).

Various lines of evidence suggest the existence of an interaction between hypothalamic orexin and mesolimbic DA systems (36–38). Thus, the expression of both orexin receptors in the hypothalamic and mesolimbic regions (39–41) suggests that orexins regulate food intake by interacting with mesolimbic DA neurons.

Orexin neurons strongly innervate the VTA and the NAc (42); the NAc shell receives the majority of orexin inputs with respect to the other compartment of the NAc, the core, and it is thus more involved in orexin functions (9). Moreover, the shell of the NAc and not the core, expresses orexin receptors (39, 41); this area also sends projections to LH orexin neurons (43–45). All these connections let us hypothesize that orexin neurons in the LH, dopaminergic neurons in the VTA, and the NAc shell cooperate in the reward processes. Thus, it has been hypothesized that orexin exerts an activation of DA neurons in the VTA directly (46) by suppressing the GABAergic (47) and/or by enhancing the glutamatergic input on DA neurons (5, 48).

With the aim of clarifying whether orexin can affect the responsiveness of mesolimbic DA transmission during feeding, we analyzed the effect of OxA on basal DA release in the NAc shell and its responsiveness to food consumption by using the microdialysis technique in freely moving rats.

OxA is a large peptide and does not cross the blood–brain barrier (BBB) (49, 50). Therefore, in this work, OxA has been incorporated into liposomes whose surface has been decorated with the anti-transferrin-monoclonal antibody OX26 (OX26-mAb) or lactoferrin. Indeed, it has been demonstrated that the interaction of OX26-mAb with the transferrin receptor or the interaction of lactoferrin with lactoferrin receptor can promote the accumulation of the modified liposomes and of the carried drug into the CNS parenchyma through receptor-mediated transcytosis (RMT) (51). However, the lactoferrin covalently bound to the vesicle's surface must compete with the endogenous lactoferrin for the same lactoferrin receptor-binding site, while OX26-mAb does not compete with the endogenous transferrin because it binds to another transferrin receptor-binding site. Because of this possible different binding kinetic rate, we wanted to test if the two liposome formulations have a different influence on the delivery of OxA to the CNS parenchyma (and on its ability to affect the responsiveness of mesolimbic DA transmission during feeding). Here we show the effect of the systemic administration of orexin A loaded liposomes on DA release in the NAc shell and its responsiveness to sucrose feeding.

Moreover, in order to investigate the role of OxR1 in the action of orexin on DA transmission, we administered an OxR1 antagonist (SB 334867) 30 min before orexin injection.

## Materials and methods

### Animals

Male Sprague–Dawley rats (Envigo Italy, Udine, Italy) were housed in the animal facility, and standard food (MIL topi e ratti, GLP diets, Stefano Morini, S. Polo D'Enza, RE, Italy) and water were given *ad libitum*. Animals were housed under constant temperature (23°C), humidity (60%), and a 12 h light/dark cycle (light from 8.00 a.m. to 8.00 p.m.) for at least a week. The weight of the animals upon arrival was 250–275 g.

All animal experiments were conducted in accordance with the guidelines for the care and use of experimental animals of the European Communities Council (2010/63/UE L 276 20/10/2010) and with Italian law (DL: 04.03.2014, N° 26). The study was approved by the Animal Welfare Body of the University of Cagliari (OPBA) and by the Ministero della Salute (authorization n° 1152/2015-PR). All efforts have been made to minimize suffering and the number of animals used. The number of animals was calculated using the software GPower.

### Surgery

Rats were anesthetized using our previously reported method (34) and implanted with a polyethylene catheter (Portex tubing PE 0.58 × 0.190 mm, Scientific Laboratory Supplies, UK) in the right femoral vein. The catheter was tunneled subcutaneously to exit at the nape of the neck according to the method described by Pontieri et al. (52). A guide cannula of 21G aimed at the NAc shell (Plasticone, Roanoke, VA, USA) was stereotaxically and unilaterally (left or right hemisphere) implanted under the following coordinates: A: 1.8; L: 1 from bregma, V: −3.6 from dura, according to the atlas of Paxinos and Watson (53). The guide cannulae were plugged with a dummy cannula.

After the surgery, the rats were housed in individual cages (45 × 21 × 24 cm) under the same conditions mentioned above. The rats were left to recover for 10 days; during this period, the catheters were flushed daily with heparinized saline (heparin 250 U/ml in 0.9% sterile saline) and with 0.1 ml of gentamicin (40 mg/ml) for 5 days. The rats were handled once a day for 5 min during the entire training period.

The rats were fed 10 g of sucrose pellets 6 days before the microdialysis experiment to avoid having neophobia.

Standard food (MIL topi e ratti, GLP diets, Stefano Morini, S. Polo D'Enza, RE, Italy) and water were available *ad libitum*.

### Microdialysis

#### Probe preparation

Microdialysis probes were prepared according to the method described by Lecca et al. (54) and by us (33) by using AN69 membrane (Hospal Dasco, Italy). The dialyzing portion of the probe was 1.5 mm long.

#### Microdialysis experiments

**Figure 1:**
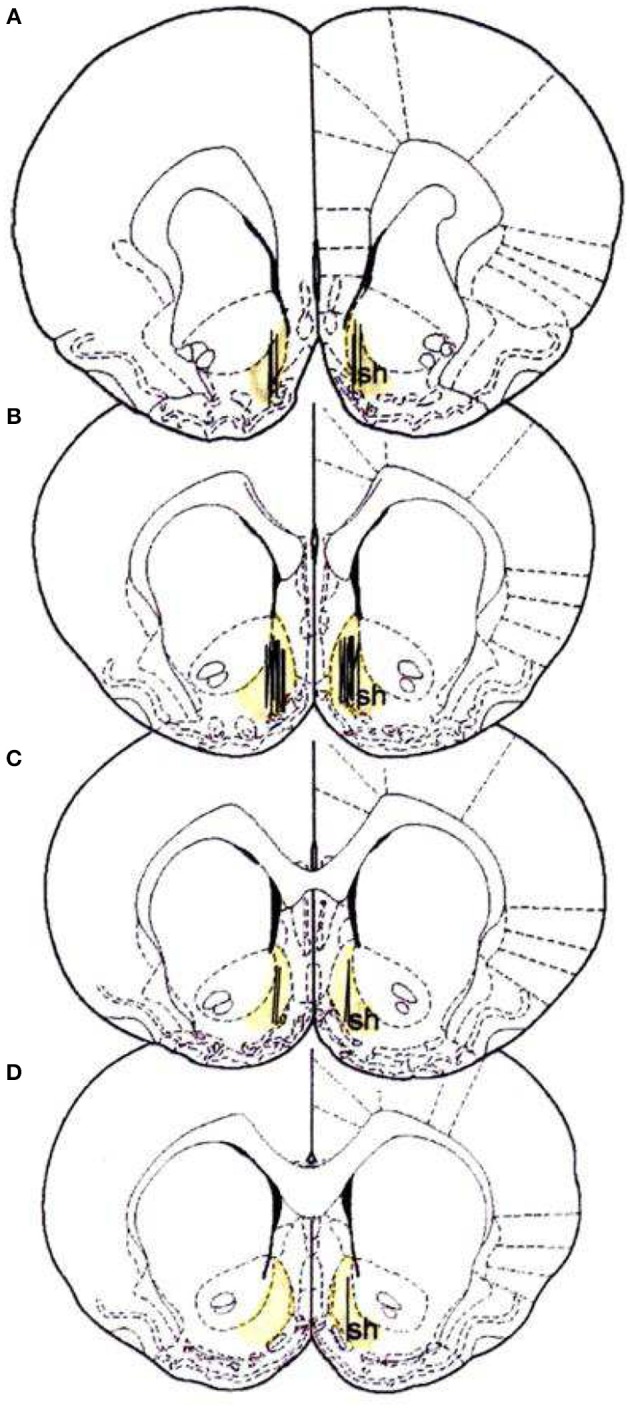
Localization of dialysis portion within the NAc shell; sh, shell (53). **(A)** +2.2 anterior to bregma (22 rats), **(B)** +1.7 anterior to bregma (34 rats), **(C)** +1.6 anterior to bregma (14 rats), **(D)** +1.2 anterior to bregma (3 rats).

On the day before the microdialysis experiment, the rats were brought in the microdialysis room and placed in large hemispheric bowls (diameter, 50 cm) with bedding on the bottom, where they spent the night with water and standard food *ad libitum*. At the beginning of the session, the probes were connected to an infusion pump and perfused with Ringer's solution [147 mM NaCl, 4 mM KCl, 2.2 mM CaCl_2_; the use of 2.2 mM Ca^2+^ in the Ringer can be referenced to the study by Lecca et al. (55)] at a constant rate of 1 μl/min. The dummy cannula was removed, and the microdialysis probe was inserted through the guide cannula. The microdialysis probe comes out from the cannula for 4 mm into the brain tissue. The final coordinates of the microdialysis probe inserted in the NAc shell were as follows: A:1.8; L: 1 from bregma, V: −7.6 from dura. The location of the probes is reported in Figure 1.

After 10 min, the dialysate samples (5 μl) began to be collected every 5 min and were injected without being purified into a high-performance liquid chromatograph (HPLC) equipped with a reverse-phase column (LC-18 DB, 15 cm, 5 μm particle size, Supelco) and a coulometric detector (ESA, Coulochem II, Bedford, MA) to quantify DA. The first and second electrodes of the detector were set at +125 (oxidation) and −175 mV (reduction). The mobile phase comprised 50 mM NaH_2_PO_4_, 0.1 mM Na_2_-EDTA, 0.5 mM n-octyl sodium sulfate, and 15% (v/v) methanol and had pH of 5.5 (obtained by adding Na_2_HPO_4_). Under these conditions, the sensitivity of the assay for DA was 5 fmol/sample.

Microdialysis samples were also analyzed by injection into an ultra-HPLC (UHPLC, ALEXYS Neurotransmitter analyzer, Antec) equipped with a column NeuroSep (C18 110, 1.0 × 100 mm, 1.7 μm) and an electrochemical amperometric detector (DECADE II SCC). The mobile phase comprised 100 mM phosphoric acid, 100 mM citric acid, 0.1 mM EDTA, and acetonitrile (8% v/v), 3 mM. Under these conditions, the sensitivity of the assay for DA was 5 fmol/sample.

Basal dialysate DA was calculated as the mean of the last three consecutive samples, which differed by no more than 10% and collected before the experimental session.

At the end of each microdialysis session, the probe was removed and the guide cannula was plugged again with a sterilized dummy cannula.

### Drugs and food

#### OxA

OxA was purchased from Tocris Bioscience (UK) and dissolved in saline (NaCl 0.9%, w/v) to obtain a final concentration of 292 μg/ml. The solution was divided in 1.5 ml conical microtubes and stored at −20°C until use for liposome preparation. OxA loaded liposomes were indispensable because the OxA does not cross the BBB (49, 50). In this study we used the procedure reported by De Luca et al. (51) for the study of the central effects of senktide, a peptide that also does not cross the BBB.

#### Liposome preparation

Distearoylphosphatidylcholine (DSPC) and polyethylene glycoldistearoylphosphatidylethanolamine-(DSPE-PEG) were purchased from Lipoid (Ludwigshafen, Germany). DSPE-PEG–maleimide was purchased from NOF Corporation (Tokyo, Japan). Cholesterol, sepharose CL-4B, and 2-iminothiolane (Traut's reagent) were obtained from Sigma (Milan, Italy). Centriprep-30 (molecular weight cut-off: 30,000) concentrators were acquired from Amicon (Billerica, MA). The anti-rat transferrin receptor OX26-mAb was supplied by AbD Serotec (Kidlington, UK).

OX26-mAb- and lactoferrin-conjugated liposomes (LIPO-OX26 and LIPO-lact) were prepared using thin-film hydration. For 1 ml of liposomal dispersion, DSPC (5.2 mmol), cholesterol (4.5 mmol), DSPE-PEG (0.3 mmol), and DSPE-PEG–maleimide (0.18 mmol) were dissolved in a chloroform/methanol (2:1 v/v) mixture. The solvent was evaporated under reduced pressure at room temperature. The obtained lipid film was hydrated under mechanical stirring with saline (vehicle, unloaded liposomes) or with saline containing OxA (292 μg/ml) (OxA loaded liposomes) at 65°C. The obtained liposomes were subjected to sonication using a Soniprep 150 ultrasonic disintegrator (MSE Crowley, London, United Kingdom) until a clear opalescent dispersion was obtained. For preparation of immunoliposomes (LIPO-OX26), OX26-mAb (1 mg; 0.0058 mmol) was thiolated by reaction with iminothiolane (32 mg; 0.23 mmol) in 3 ml of borate buffer solution adjusted at pH 8.1. EDTA solution (4 mM) was added to chelate divalent metals present in the solution. The mixture was stirred for 2 h at room temperature. Thiolated OX26-mAb solutions were concentrated [the buffer replaced with phosphate buffer solution (pH 7.4)] by using a Centriprep-30 concentrator (molecular weight cut-off: 30,000). Finally, the purified thiolated OX26-mAb was incubated with maleimide-grafted liposomes overnight at room temperature. Lactoferrin-conjugated liposomes (LIPO-lact) were prepared with the same method using iminothiolane and lactoferrin concentration of 0.09 mmol. Immunoliposomes and lactoferrin-conjugated liposomes were separated from unentrapped OxA and free OX26-mAb or lactoferrin by gel filtration chromatography (Sepharose CL-4B) using saline as the eluent.

Two different OxA loaded liposomes preparation were obtained: OxA loaded immunoliposomes (OxA-LIPO-OX26) and OxA loaded lactoferrin-conjugated liposomes (OxA-LIPO-lact).

As a liposome preparation control, two different unloaded liposomes (vehicle) were obtained: unloaded immunoliposomes and unloaded Lactoferrin-conjugated liposomes.

The loaded and unloaded liposome preparations were administered intravenously (1 ml/Kg).

#### HPLC-DAD analysis

Before HPLC injection, liposome samples containing OxA were diluted 1:1 (v/v) with methanol. The gradient profile for the analysis of OxA was as follows: initial 10:90 (v/v) acetonitrile/water, reaching 40:60 (v/v) in 10 min. The injection volume was 20 μl, and the flow rate was 0.6 ml/min. The analysis was performed at the wavelength of 215 nm by using a Phenomenex Sinergi 4μ C18 column (150 × 4.60 mm, 4 μm). The retention time for OxA was 7.68 min. The amount of OxA was 60 μg/ml.

#### SB 334768

The OxR1 antagonist, SB 334867 (Tocris Bioscience, UK), was dissolved in 1% (2-hydroxypropyl)-β-cyclodextrin (Sigma) and 10% DMSO in 90% distilled water (56) and intraperitoneally (ip) administered to the rats in a volume of 3 ml/kg body weight at a dose of 30 mg/kg.

#### Sucrose

Sucrose pellets (45 mg each) were utilized as palatable food (Test Diet, 1050 Progress Drive, Richmond, IN 47374).

### Experimental groups

#### Experiment 1: Effect of free OxA, OxA-loaded LIPO-OX26, OxA-loaded LIPO-Lact, and OxR1 antagonist on NAc shell DA transmission

**Figure 2:**
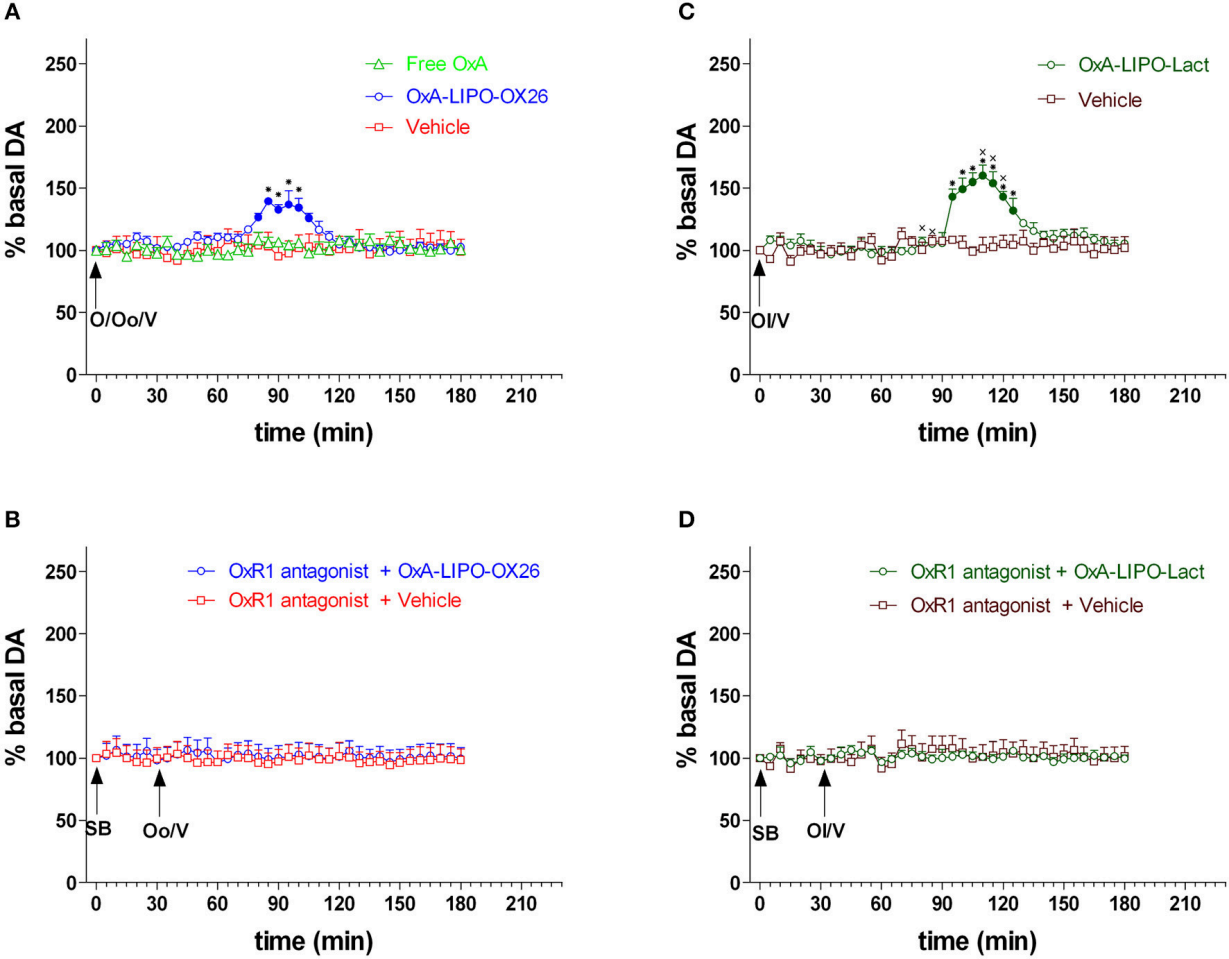
Effect of free OxA, OxA-LIPO-OX26, OxA-LIPO-Lact, and of OxR1 antagonist on NAc shell DA transmission. **(A)** Modification of DA concentration after free OxA (O), OxA-LIPO-OX26 (Oo), and vehicle (V) administration. **(B)** Effect of OxR1 antagonist (SB) on DA transmission after OxA-LIPO-OX26 (Oo) and vehicle (V) administration. **(C)** Modification of DA concentration after OxA-LIPO-Lact (Ol) and vehicle (V) administration. **(D)** Effect of OxR1 antagonist (SB) on DA transmission after OxA-LIPO-Lact (Ol) and vehicle (V) administration. Data are presented as means ± SEM of the results, expressed as a percentage of basal DA, obtained in the 40 rats. Empty symbols: *p* > 0.05 vs. basal values; Filled symbols: *p* < 0.05 vs. basal values; ^*^p < 0.05 with respect to free OxA, vehicle groups and with respect to the OxR1 antagonist treated rats. ^x^*p* < 0.05 with respect to the OxA-LIPO-OX26 group.

The rats were treated with peptide-loaded liposomes conjugated to OX26-mAb (OxA-LIPO-OX26, 60 μg/kg/ml iv; *N* = 8) (Figure 2A) or with OxA-loaded lactoferrin-conjugated liposomes (OxA-LIPO-lact, 60 μg/kg//ml iv; *N* = 4) (Figure 2C) in order to determine the effect of OxA on NAc shell DA transmission. The control groups received 1 ml/kg iv of free OxA (60 μl/Kg/ml) (*N* = 4), empty LIPO-OX26 (*N* = 4), or empty LIPO-lact (*N* = 4) (vehicle, 1 ml/Kg iv) (Figures 2A,C). Other groups of animals were injected with the OxR1 antagonist (30 mg/kg/3 ml i.p.) 30 min before intravenous administration of empty or OxA-LIPO-OX26 (*N* = 8) (Figure 2B) and empty or OxA-LIPO-lact (*N* = 8) (Figure 2D) in order to evaluate the role of OxR1 in the increased NAc shell DA concentration induced by OxA.

#### Experiment 2: Responsiveness of NAc shell DA during sucrose pellet consumption and number of sucrose pellets eaten after OxA-LIPO-OX26 and OxA-LIPO-lact

**Figure 3:**
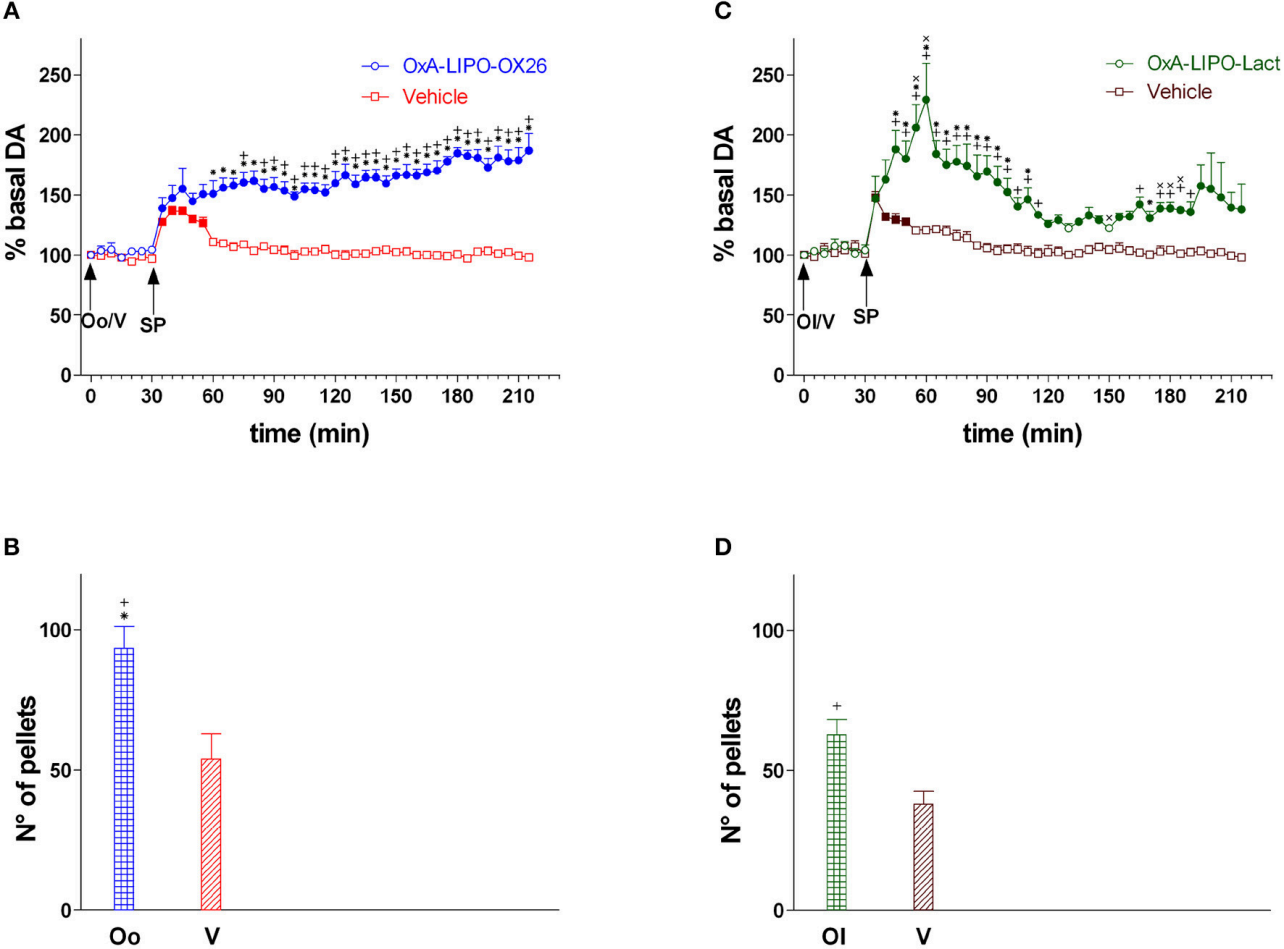
Responsiveness of NAc shell DA during sucrose pellet consumption and number of sucrose pellets eaten after OxA-LIPO-OX26, OxA-LIPO-Lact, and effect of OxR1 antagonist pretreatment. **(A)** Modification of DA concentration after OxA-LIPO-OX26 (Oo) and vehicle (V) administration and subsequent sucrose pellets consumption. **(B)** Number of sucrose pellets eaten by rats pretreated with OxA-LIPO-OX26 (Oo) or with vehicle (V). **(C)** Modification of DA concentration after OxA-LIPO-Lact (Ol) and vehicle (V) administration and subsequent sucrose pellets consumption. **(D)** number of sucrose pellets eaten by rats pretreated with OxA-LIPO-Lact (Ol) or with vehicle (V). Data are presented as means ± SEM of the results, expressed as a percentage of basal DA, and of the n° of sucrose pellets eaten, obtained in 21 rats. Empty symbols: *p* > 0.05 vs. basal values; Filled symbols: *p* < 0.05 vs. basal values; ^*^p < 0.05 vs. values obtained in the vehicle group (empty LIPO-OX26). ^+^*p* < 0.05 with respect to the vehicle group (empty LIPO-Lact). ^x^*p* < 0.05 with respect to the OxA-LIPO-OX26 group.

In this experiment, the role of OxA on food consumption and NAc shell DA responsiveness during feeding was tested. The animals were treated with empty LIPO-OX26 (*N* = 5) (vehicle group, 1 ml/kg iv) or OxA- LIPO-OX26 (*N* = 6) (60 μg/kg/ml iv) or with empty LIPO-lact (*N* = 5) (vehicle group, 1 ml/kg iv) or OxA-LIPO-lact (60 μg/kg/ml iv) (*N* = 5) and fed with sucrose pellets (Figures 3A–D) in order to clarify the role of OxA on food consumption and NAc shell DA responsiveness during feeding. The number of eaten sucrose pellets was recorded.

#### Experiment 3: Effect of OxR1 antagonist on NAc shell DA transmission during sucrose pellet consumption and on the number of sucrose pellets eaten after OxA-LIPO-OX26 or OxA-LIPO-lact

**Figure 4:**
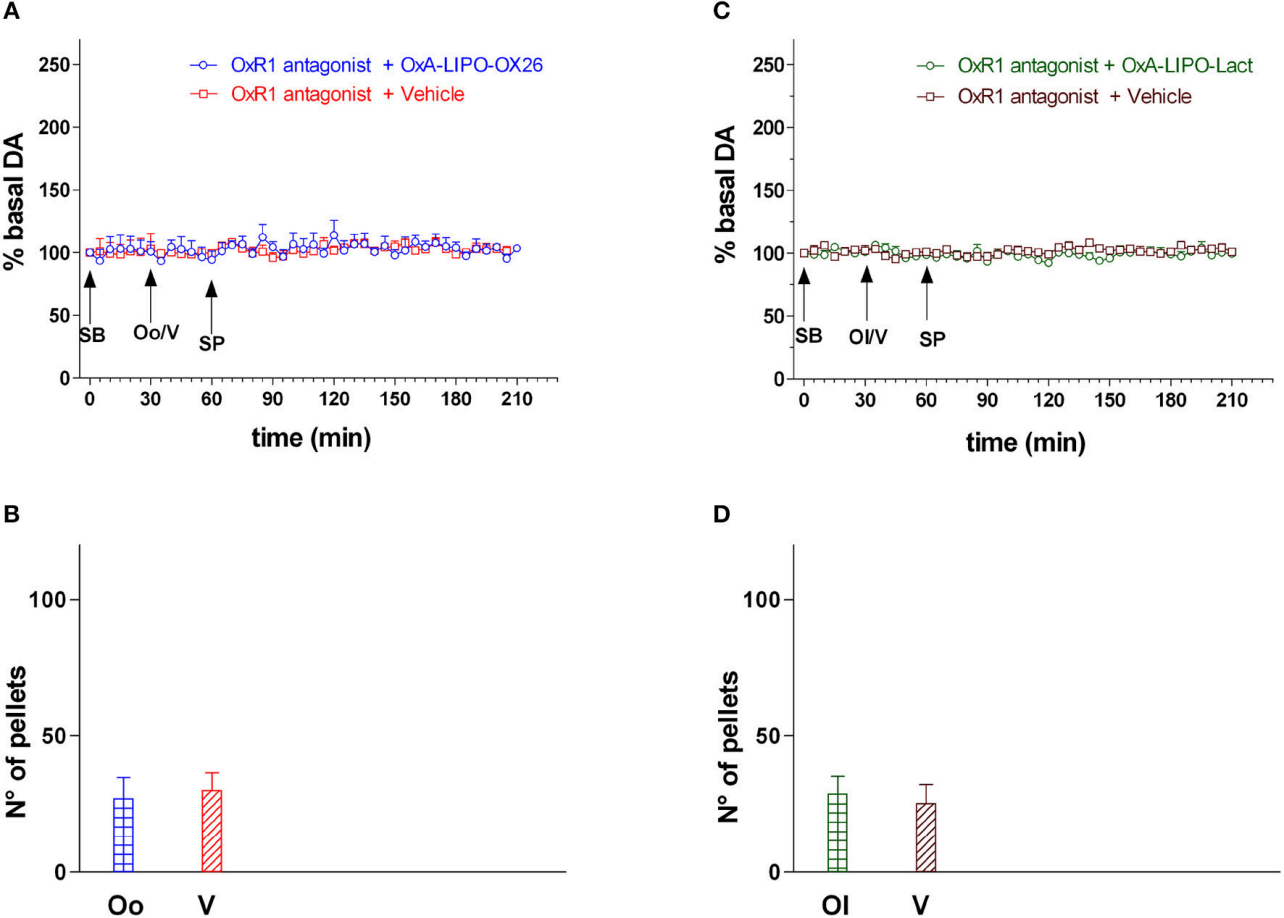
Effect of OxA-LIPO-OX26, OxA-LIPO-Lact and of OxR1 antagonist on NAc shell DA transmission and number of sucrose pellets eaten. **(A)** Modification of DA concentration after OxR1 antagonist (SB) and OxA-LIPO-OX26 (Oo) or vehicle (V) administration and subsequent sucrose pellets consumption. **(B)** Number of sucrose pellets eaten by rats pretreated with OxR1 antagonist (SB) and with OxA-LIPO-OX26 (Oo) or with vehicle (V). **(C)** Modification of DA concentration after OxR1 antagonist (SB) and OxA-LIPO-Lact (Ol) or vehicle (V) administration and subsequent sucrose pellets consumption. **(D)** Number of sucrose pellets eaten by rats pretreated with OxR1 antagonist (SB) and with OxA-LIPO-Lact (Ol) or with vehicle (V). DA data are presented as means ± SEM of the results, expressed as a percentage of basal DA, and of the n° of sucrose pellets eaten, obtained in 16 rats. Empty symbols: *p* > 0.05 vs. basal values.

The animals were injected with the OxR1 antagonist (30 mg/kg/3 ml i.p.) 30 min before intravenous administration with empty LIPO-OX26 (*N* = 4) (vehicle, 1 ml/kg iv) or OxA-LIPO-OX26 (*N* = 4) (60 μg/kg/ml iv) and with empty LIPO-lact (*N* = 4) (vehicle, 1 ml/kg iv) or OxA-LIPO-lact (*N* = 4) (60 μg/kg/ml iv) in order to test the role of OxR1 in strengthening the increase in NAc shell DA induced by OxA after sucrose pellet consumption. The effect of OxR1 antagonist on the amount of eaten pellets was also studied (Figures 4A–D).

### Histology

At the end of all experimental procedures, the rats were anesthetized as previously reported (34), and the brains were removed and postfixed for 5 days. The brains were cut in 100 μm-thick serial coronal slices by using a Vibratome (Technical Products International, Saint Louis MO, USA) to establish the location of the dialysis probes. The location of the probes was reconstructed and referred to the atlas of Paxinos and Watson [(53); Figure 1].

### Statistical analysis

Statistical analysis was carried out by Statistica for Windows. Basal dialysate DA concentration was calculated as the mean of the last three consecutive samples differing by no more than 10%. Changes in dialysate DA were expressed as the percent of the respective baseline values and analyzed by two or three-way ANOVA for repeated measures. The number of sucrose pellets eaten during the experiments was assessed by one-way ANOVA. Results from treatments showing significant overall changes were subjected *post-hoc* to Tukey's test, with *p* < 0.05 as statistically significant.

## Results

Basal values of dialysate DA (fmoles/sample, means ± SEM) corresponded to 25 ± 2 (*N* = 73) in the shell of the NAc. One-way ANOVA did not show any difference between groups [*F*_(15,57)_ = 0.17; *p* = 1].

### Experiment 1: Effect of free OxA, OxA-LIPO-OX26, OxA-LIPO-lact, and OxR1 antagonist on NAc shell DA transmission

Figure 2 shows the changes in NAc shell dialysate DA after IV administration of free OxA (60 μl/Kg/ml), empty LIPO-OX26 (vehicle group) and OxA-LIPO-OX26 (60 μg/kg iv) (Figure 2A), empty LIPO-lact (vehicle group) or OxA-LIPO-Lact (60 μg/kg iv) (Figure 2C), and the effect of pretreatment with the OxR1 antagonist (30 mg/kg i.p.) administered 30 min before (Figures 2B,D).

Two-way ANOVA of DA in the dialysate samples after OxR1 antagonist administration did not show an increase in basal DA nor any difference between the four groups [*F*_(group3,12)_ = 1.0; *p* = 0.4, *F*_(time6,72)_ = 2.0; *p* = 0.07, *F*_(groupxtime18,72)_ = 0.4; *p* = 1.0].

Two-way ANOVA of DA in the dialysate samples after free OxA, OxA-LIPO-OX26, and OxA-LIPO-Lact showed an effect of OxA treatment [*F*_(2, 13)_ = 7.45; *p* < 0.001] and time [*F*_26,338_ = 14.7; *p* < 0.001] and an interaction OxA treatment × time [*F*_52,338_ = 7.63; *p* < 0.001]. *Post-hoc* testing revealed a stimulation of DA transmission only when the OxA was delivered by liposomes.

Three-way ANOVA of DA in the dialysate samples after OxA-loaded liposomes and vehicles showed an effect of OxA treatment [*F*_3,28_ = 7.4; *p* < 0.001], OXR1 antagonist pretreatment [*F*_1,28_ = 18.5; *p* < 0.001], and time [*F*_24,672_ = 8.9; *p* < 0.001] and the interactions of OxA treatment × OXR1 antagonist pretreatment [*F*_3,28_ = 3.3; *p* = 0.03], OxA treatment × time [*F*_72,672_ = 5.3; *p* < 0.001], OXR1 antagonist pretreatment × time [*F*_24,672_ = 11.3; *p* < 0.001], and OxA treatment × OXR1 antagonist pretreatment × time [*F*_72,672_ = 4.8; *p* < 0.001]. A *post-hoc* Tukey's test revealed an increase in DA in the NAc shell of rats administered IV with OxA-LIPO-OX26 and OxA-LIPO-Lact compared with that in the control rats treated with empty liposome (Figures 2A,C). *Post-hoc* testing also revealed that the increase of DA was higher and delayed in the animals treated with OxA-LIPO-Lact compared with the animals treated with OxA-LIPO-OX26. The increase was completely prevented by IP injection of the OXR1 antagonist (Figures 2B,D).

### Experiment 2: Responsiveness of NAc shell DA during sucrose pellet consumption and number of sucrose pellets eaten after OxA-LIPO-OX26 and after OxA-LIPO-lact

Figure 3 shows the time-course of dialysate DA in the NAc shell during sucrose pellet consumption and the number of eaten sucrose pellets (bars) after IV administration of empty liposomes (vehicle), OxA-LIPO-OX26 (60 μg/kg iv), or OxA-LIPO-Lact (60 μg/kg iv).

Two-way ANOVA of DA in the dialysate samples after OxA-LIPO-OX26, OxA-LIPO-Lact, or vehicle administration did not show an increase in basal DA nor any difference between the four groups [*F*_(group3,17)_ = 0.74; *p* = 0.5, *F*_(time6,102)_ = 0.4; *p* = 0.9, *F*_(groupxtime18,102)_ = 0.9; *p* = 0.5].

Two-way ANOVA of DA in the dialysate samples after sucrose pellet consumption subsequent to the OxA (LIPO-OX26 and LIPO-Lact) or vehicle treatment showed an effect of OxA treatment [*F*_3,17_ = 48.15; *p* < 0.001] and time [*F*_32,544_ = 3.28; *p* < 0.001] and an interaction of OxA treatment × time [*F*_96,544_ = 6.1; *p* < 0.001]. *Post-hoc* analysis revealed a strengthening of the increase in DA induced by sucrose pellet consumption in rats pretreated with the two different OxA liposomes compared to the vehicle groups.

One-way ANOVA of sucrose pellets eaten by the four groups of rats showed that animals pretreated with OxA consumed more sucrose pellets than the vehicle groups [*F*_3,17_ = 11.84; *p* < 0.001].

### Experiment 3: Effect of OxR1 antagonist on NAc shell DA transmission during sucrose pellet consumption and on the number of sucrose pellets eaten after OxA-LIPO-OX26 and after OxA-LIPO-lact

Figure 4 shows the DA responsiveness of the NAc shell after administering the OXR1 antagonist (30 mg/kg i.p.) and during sucrose pellet consumption and the number of eaten sucrose pellets (bars) in rats treated with the empty liposomes (vehicle), OxA-LIPO-OX26 (60 μg/kg iv), or OxA-LIPO-Lact (60 μg/kg iv) 30 min after OxR1 antagonist.

Two-way ANOVA of DA in the dialysate samples after OxR1 antagonist administration did not show an increase in basal DA nor any difference between the four groups [*F*_(group3,12)_ = 0.42; *p* = 0.7, *F*_(time6,72)_ = 0.74; *p* = 0.6, *F*_(groupxtime18,72)_ = 0.63; *p* = 0.9].

Two-way ANOVA of DA in the dialysate samples after OxA-LIPO-OX26, OxA-LIPO-Lact, or vehicle administration did not show an increase in basal DA nor any difference between the four groups [*F*_(group3,12)_ = 0.33; *p* = 0.8, *F*_(time6,72)_ = 0.68; *p* = 0.7, *F*_(groupxtime18,72)_ = 1.24; *p* = 0.3].

Two-way ANOVA of DA in the dialysate samples during sucrose pellet consumption after SB administration did not show an increase in basal DA nor any difference between the two groups [*F*_(group3,12)_ = 1.9; *p* = 0.19, *F*_(time24,288)_ = 0.9; *p* = 0.58, *F*_(groupxtime72,288)_ = 0.56; *p* = 1.0].

One-way ANOVA of sucrose pellets eaten by the two groups of rats did not show any difference [*F*_3,12_ = 1.1; *p* = 0.4].

Three-way ANOVA of DA in the dialysate samples during sucrose pellet consumption in rats of experiment 2 and experiment 3 revealed an effect of OxA treatment [*F*_3,29_ = 32.23; *p* < 0.001], OXR1 antagonist pretreatment [*F*_1,29_ = 157.5; *p* < 0.001], and time [*F*_32,928_ = 6.38; *p* < 0.001] and the interactions of OxA treatment × OXR1 antagonist pretreatment [*F*_3,29_ = 33.26; *p* < 0.001], OxA treatment × time [*F*_96,928_ = 3.62; *p* < 0.001], OXR1 antagonist pretreatment × time [*F*_32,928_ = 7.49; *p* < 0.001], and OxA treatment × OXR1 antagonist pretreatment × time [*F*_96,928_ = 3.9; *p* < 0.001]. *Post-hoc* testing showed that the OxR1 antagonist treatment completely prevented the effect of OxA given by the two different liposomes on the increase in DA induced by sucrose pellet consumption and completely prevented any effect of sucrose pellets on DA transmission in control animals.

Two-way ANOVA of sucrose pellets eaten by the rats from experiments 2 and 3 showed an effect of OxA treatment [*F*_3,29_ = 8.16; *p* < 0.001], OXR1 antagonist pretreatment [*F*_1,29_ = 64.0; *p* < 0.001], and the interactions of OxA treatment × OXR1 antagonist pretreatment [*F*_3,29_ = 7.8; *p* < 0.001]. *Post-hoc* testing revealed that OxR1 antagonist treatment reduced the number of eaten pellets in both groups of OxA and vehicle treated animals.

## Discussion

The most important result of the present study is that the peptide OxA, which was systemically administered by liposomes conjugated to specific homing devices (anti-transferrin-monoclonal antibody or lactoferrin), increased the dialysate DA in the NAc shell, strongly potentiated the DA response to food, and increased the number of sucrose pellets eaten by awake and freely moving rats. These effects on DA transmission were completely blocked, and the number of sucrose pellets consumed was reduced by the OXR1 antagonist SB 334867 (30 mg/kg IP). Intravenous administration of 60 μg/kg/ml free OxA (i.e., not incorporated into liposomes) failed to modify NAc shell dialysate DA.

Experimental and correlative evidence has been provided with an activation of DA transmission by OxA administration. Thus, OxA infusion into VTA stimulates DA transmission in the NAc (57). Borgland et al. (5) reported that OxA may be released into the VTA, stimulating DA transmission in the VTA terminal regions (57–59). Furthermore, direct monitoring by cerebral microdialysis in freely moving animals of DA concentration in the NAc shell after systemic OxA administration was not still investigated.

In the present study we analyzed the effect of OxA on basal DA transmission in the NAc shell and on DA modification during feeding. For the first time OxA has been administered systemically loaded in lactoferrin- and antitransferrin-modified liposomes. After intravenous injection, these nanocarriers deliver the OxA into the CNS, allowing the abandonment of the intracranial administration routes that are highly invasive and not easy to perform.

The administration of OxA OX26-modified liposomes increased DA release in the NAc shell from 80 to 110 min after the injection (Figure 2A). The increase caused by OxA administration by lactoferrin-conjugated liposomes induced a delayed increase in DA, peaking from 95 to 125 min after IV injection (Figure 2B). The difference in time course could be explained by considering that lactoferrin- and OX26-modified liposomes bind to different brain capillary endothelial receptors (lactoferrin and transferrin receptors, respectively) at different binding kinetic rates. Moreover, lactoferrin-modified liposomes compete with endogenous lactoferrin to bind to the lactoferrin receptor, whereas OX26-modified liposomes bind to a different site from that of transferrin. The results obtained on the modification of DA release demonstrated that, although with a different time course, both the nanocarriers are valid systems to deliver OxA into the brain.

The increase in the NAc shell DA by OxA administration was completely blocked by intraperitoneal administration of the OXR1 antagonist SB 334867 (30 mg/kg). Furthermore, we demonstrated that SB334867 completely prevented the increase in NAc shell DA during sucrose consumption and reduced the number of sucrose pellets eaten. These results confirm the implication of OXR1 in the effect of OxA on tonic DA transmission and DA responsiveness to food. The receptor 1 for OxA is coupled to Gq proteins (60) and its activation by OxA resulted in the activation of phospholipase C. This phosphodiesterase hydrolyzes the phosphatidylinositol 4,5 biphosphate producing triphosphate inositol and diacylglycerol, two second messengers that induce an activation of neuronal cells. The activation of OxR1 on DA VTA neurons by OxA induces a stimulation of these neurons that releases DA in the NAc shell terminal area. During sucrose feeding, OxA acting on VTA OxR1 can facilitate sucrose-stimulated NAc shell DA transmission directly by increasing the basal activity of VTA DA neurons that send their projections to the NAc shell.

Our results are also consistent with reports that intracerebral administration of OxA induces feeding and increases the consumption of high-fat diet (10, 13–15). Orexin also promotes food seeking motivated by hunger (17) and by Pavlovian cues (18–20). The OxR1 antagonist, namely SB 334867, reduced food consumption (21) and chocolate or sucrose self-administration (5, 6).

The originality of this study on the effect of OxA on DA transmission is the combination of behavioral observations during feeding with neurochemical analysis in the brain of awake rats by using microdialysis techniques and systemic administration of OxA by lactoferrin- or antitransferrin-modified liposomes.

The results obtained confirm the existence of an excitatory effect of OxA on mesolimbic DA transmission, in particular the NAc shell, leading us to conclude that these two molecules can cooperate in their responsiveness to food rewards, as hypothesized by other authors (61–64).

## Conclusions

The present study demonstrated the facilitatory effect of OxA on basal DA transmission and on strengthening the DA response during sucrose consumption. We hypothesize that during feeding, hypothalamic orexinergic neurons release OxA in the VTA; OxA interacts with OxR1 located on VTA dopaminergic neurons promoting DA release in the terminal areas, such as the NAc shell. As previously reported by us DA transmission in this area plays an important role in associative reward-related learning (2) and can promote the maintenance of motivated behavior for food seeking and food intake.

Further investigations are necessary to evaluate if a hyperstimulation of orexinergic system can potentiate the mesolimbic DA response to food during eating disorders, such as binge eating or bulimia nervosa. The strengthened and continuous increase of DA in the NAc shell produces an abnormal and pathological associative learning (65, 66), which could be one of the mechanisms that underlie food addiction and overconsumption of palatable food.

## Author contributions

VB and FL conceived the study and designed the experiment. VB, FL, and AF set up the experimental procedure. VB, FL, FlC, RF, FrC, and PC conducted the experiment. FL, PC, and VB analyzed the data. VB drafted the manuscript. All authors contributed to and approved the final draft of the manuscript.

### Conflict of interest statement

The authors declare that the research was conducted in the absence of any commercial or financial relationships that could be construed as a potential conflict of interest.
